# Analysis of the Effect of Catalytic Additives in the Agricultural Waste Combustion Process

**DOI:** 10.3390/ma15103526

**Published:** 2022-05-13

**Authors:** Tomáš Najser, Błażej Gaze, Bernard Knutel, Adam Verner, Jan Najser, Marcel Mikeska, Jerzy Chojnacki, Ondřej Němček

**Affiliations:** 1 ENET Centre, VSB—Technical University of Ostrava, 17 Listopadu 2172/15, 708 00 Ostrava, Czech Republic; adam.verner@vsb.cz (A.V.); jan.najser@vsb.cz (J.N.); marcel.mikeska@vsb.cz (M.M.); ondrej.nemcek@vsb.cz (O.N.); 2 Institute of Agricultural Engineering, Wroclaw University of Environmental and Life Sciences, 51-630 Wroclaw, Poland; blazej.gaze@upwr.edu.pl (B.G.); bernard.knutel@upwr.edu.pl (B.K.); 3 Nanotechnology Centre, VSB—Technical University of Ostrava, 17 Listopadu 2172/15, 708 00 Ostrava, Czech Republic; 4 Faculty of Mechanical Engineering, Koszalin University of Technology, Racławicka Str. 15-17, 75-620 Koszalin, Poland; jerzy.chojnacki@tu.koszalin.pl

**Keywords:** agricultural waste, catalytic additives, combustion, emission reduction

## Abstract

This paper presents the research results of the effect of using calcium oxide and potassium permanganate on the combustion of pellets from wheat bran and beet pulp. The measurements were performed in the technical laboratory of the Centre of Energy Utilization of Non-Traditional Energy Sources in Ostrava. The research examined the effect of the use of chemical substances on the amount of air pollutants from biomass thermal conversion in a low-power boiler and the process temperature. First, we performed technical and elementary analyses of agricultural waste. The raw material was then comminuted, mixed with a selected additive, pelletized, and finally burned in a low-power boiler. The additive was added in three proportions: 1:20, 1:10, and 1:6.67 (i.e., 15%) relative to the fuel weight. The combustion process efficiency was measured using a flue gas analyzer and three thermocouples attached to the data recorder. From the measurement results, we were able to determine the percentage reduction of pollutant emissions into the atmosphere (CO, NO_x_, and SO_2_) due to the use of additives. Because emission standards are becoming increasingly stringent and fuel and energy prices are rising, the results presented in this article may be useful to agri-food processing plants that want to manage these materials thermally.

## 1. Introduction

The use of biomass in the energy sector can potentially reduce the emission of harmful substances into the air. It is assumed that the absorbed amount of CO_2_ during plant growth balances the amount generated in the process of biomass combustion [1]. Biomass is therefore considered a zero-emission fuel. This assumption is incorrect because burning biomass also produces other pollutants that the plant cannot absorb during the growing season. The pollutants emitted during the combustion process can be divided into primary and secondary groups. Primary pollutants are emitted into the atmosphere directly at the locations where they are produced. Primary pollutants remain in the atmosphere unchanged from the moment they are generated. The sources of primary pollution are power plants and heating devices for single-family houses. Secondary pollutants, however, are the products of physical changes and chemical reactions between the components of the atmosphere and the pollutants that flow into it. Continuous technological progress, new branches of industry, and the development of transport contribute to the increase in the number of point, line, and area emitters. The constant increase in the number of sources of harmful emissions has an adverse impact on air quality. In highly developed countries, legal directives and laws specify the emission limits for individual compounds related to the type of fuel burned or the nominal power of the emitter. Therefore, the growing number of emitters is not reflected in the amount of emissions [2].

Research commissioned by the European Environment Agency (EEA) showed that air pollution is one of the leading environmental factors affecting human life and health [2]. In highly developed countries, including EU countries, appropriate legal regulations were introduced to limit the problem of harmful emissions [2,3,4]. During the combustion process, harmful substances emitted in exhaust gases include suspended dust (PM), carbon monoxide (CO), nitrogen oxides (NO_x_), and sulfur oxides (SO_x_). The hazardous compounds emitted in exhaust gases are polycyclic aromatic hydrocarbons (PAHs) and volatile organic compounds (VOCs). The emissions of these compounds are incredibly high in the case of biomass combustion [4,5,6,7,8]. Too high of a concentration in the air of the compounds mentioned above can potentially lead to deficiencies in the human circulatory and respiratory systems. Even a slight excess in the permissible concentrations of pollutants in the air may cause disturbances in concentration and perception [8,9,10,11,12].

The implemented EU Emissions Trading System (EU ETS) is one of the main tools in the fight against air pollution. This system covers approximately 40% of all greenhouse gas (GHG) emissions in the European Economic Area. The emissions trading system, in addition to new legal regulations, enables the continuous reduction of permissible limit values in reducing the total emission of pollutants [13]. According to the European Commission, all of these measures aim to bring about an economic transformation toward climate neutrality, which must be attained by 2050 [14,15].

Catalytic substances added to combusted energy carriers are a group of compounds that improve the efficiency of the combustion process. One of the tasks of catalytic additives is to increase the oxidation process of fuel particles together with products of incomplete combustion. Depending on the used catalyst, it is possible to reduce or oxidize selected harmful compounds. The use of co-combustion of active substances with fuels is able to reduce greenhouse gas emissions and eliminate carcinogenic, mutagenic, and toxic compounds [16]. Catalytic additives based on copper (Cu) and manganese (Mn) oxide, with the use of aluminum oxide (Al_2_O_2_) as a carrier, demonstrated the effect of reducing carbon monoxide (CO) emission and particulate matter during the combustion of solid biomass. The great advantage of this type of catalyst is its low price [16,17,18,19]. During our research in 2021 at the University of Life Sciences in Wrocław, we prepared five different catalytic compounds based on TiO_2_, MnO_2_, Cu (NO_3_)_2_‧3H_2_O, 8% K_2_PtCl_2_ solution, and 32% aqueous urea solution. Sodium aluminum silicate was used as the carrier for the active substance. The research indicated that the use of these catalytic additives in the combustion of sunflower husk pellets led to a reduction in the emission of harmful substances and increased the combustion temperature. The observed increase in combustion temperature caused a decrease in the carbon monoxide (CO) concentration. A reduction in the proportion of incomplete combustion in the biomass boiler indirectly contributed to a decrease in the amount of fuel burned [20].

One method for managing and eliminating harmful emissions is promoting solutions based on renewable energy in heat and electricity generation systems. Mainly in large and low-power installations, this transformation consists of replacing fossil fuels, such as coal, with high-quality biomass fuel [21].

During the combustion of biomass, the concentration of pollutants in exhaust gases is many times lower than the composition of exhaust gases from coal combustion [22,23]. Another advantage of the use of biomass energy is its local application, thereby reducing emissions produced through transportation. The use of biomass fuel produced from local raw materials also permits the diversification of energy sources, which is now a desirable operation [24,25].

Considering the current state of development in alternative energy sources, we may safely conclude that in the era of phasing out fossil fuels, fuels from biomass will soon take over the role of basic energy carrier, for example, in heat generation systems. Compared with conventional energy sources, biomass fuels are a low-emission alternative in the production of electricity and heat. Biomass with high energy potential is found in almost every corner of the world. Depending on its physicochemical properties, biomass can be transformed in thermal processes, for example, gasification, pyrolysis, and direct combustion, to exploit its energy [26].

In the area where we conducted our research, sugar production is a major industry. In the production of sugar from sugar beet, waste is generated in the form of beet pulp. The companies that produce sugar have problems managing this type of biomass waste. Previously, sugar pulp was used as feed for farm animals, but due to decreasing numbers of pig and cattle breeders in the nearest region and the transition of farms to automated feeding with ready-mixed feed, the problem of beet pulp management has become significant.

The second local biomass waste that requires management is wheat bran, which is formed during the production of wheat flour. Local companies that produce flour sell a small amount of this waste as an additive to animal feed. However, the production of waste in the form of wheat bran is greater than the market demand and generates a large quantity of biomass waste that also requires management.

This paper consists of five sections. Section 1 includes information about air pollution and its effect on health, the types of catalysts, and the methods of waste biomass energy management. Section 2 describes the methods and equipment used for measuring exhaust gas quality and the temperatures in the combustion chamber and flue gas duct. Section 3 discusses the results of our research, which investigated the effect of additives (potassium permanganate and calcium oxide) on emissions produced by biomass waste combustion. Section 4 summarizes the research outcomes. Section 5 presents conclusions drawn from the research and proposes follow-up studies in this field.

## 2. Materials and Methods

### 2.1. Research Experiment

**Figure 1:**
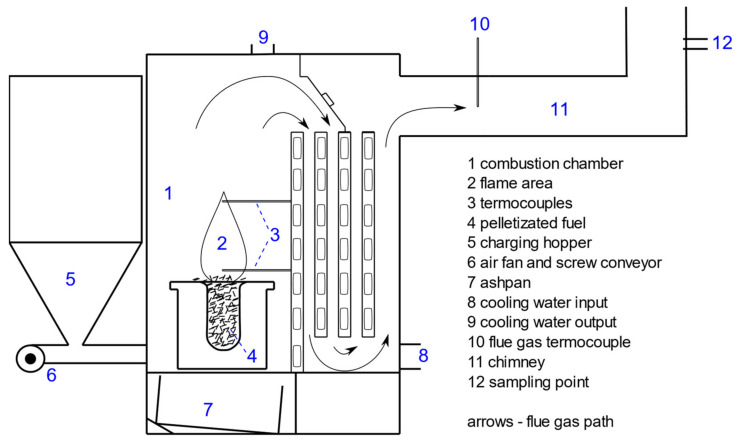
Schematic diagram of the boiler, thermocouples, and sampling points.

The study investigated the use of a low-power, fully automated retort boiler with a nominal power of 31.5 kW (VARIANT SL-33 A, produced by Slokov, Moravský Písek, Czech Republic). The operating parameters of the device were regulated using a controller. The readings of the temperature sensor and lambda probes enabled the control unit to adjust the energy carrier and air supplied to the combustion chamber to provide suitable doses. An exhaust gas fan and auger coupled with a pellet hopper regulated the air and fuel doses. The heat produced from the fuel thermal conversion process was transferred to the environment through two fan heaters with a heating capacity of 30 kW each. The combustion process was continuous, and the doses of air and fuel were injected periodically over short periods. These parameters were the same for each type of biomass fuel used in the combustion process. A schematic diagram of the boiler is presented in Figure 1, showing the locations of the thermocouples and sampling points.

The specifications of the pellet boiler are shown in Table 1.

### 2.2. Materials

**Figure 2:**
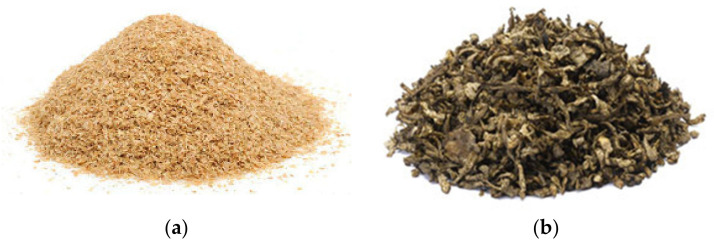
Agricultural waste used in the combustion process in the biomass low-power boiler: (**a**) wheat bran and (**b**) beet pulp.

The energy carrier was a pellet with a diameter of 6 mm and was composed of agricultural waste, i.e., wheat bran (Figure 2a) and beet pulp (Figure 2b).

**Figure 3:**
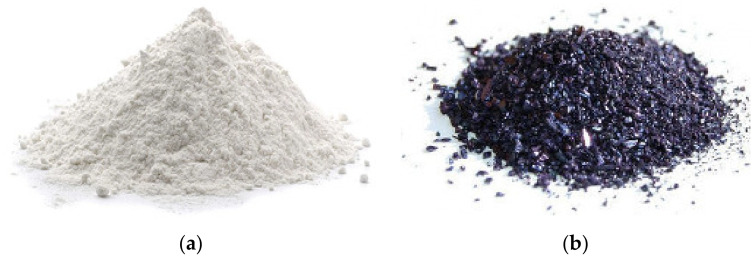
Catalytic additives: (**a**) calcium oxide and (**b**) potassium permanganate.

The thermal conversion process of the pellets included the use of catalytic additives in the form of calcium oxide CaO (Figure 3a) and potassium permanganate KMnO_4_ (Figure 3b) with purities of 99.5%. They were added to the fuel during pelletization to ground raw agricultural material in concentrations of 5, 10, and 15%.

### 2.3. Biofuel Physicochemical Analysis

All tests to analyze the physicochemical properties of the biomass materials were performed three times. Using the TGA method and a TGA 701 analyzer, we examined the biomass energy carriers to determine their moisture content, volatile substance content, ash content, and calorific values. Table 2 lists the technical specifications of the TGA 701 analyzer.

Gross calorific values were ascertained using an IKA C 200 calorimeter. A higher heating value was applied in accordance with the PN-EN standard [27]. Table 3 lists the technical specification of the IKA C 200 calorimeter.

Using a PerkinElmer CHNS/O 2400 analyzer (Waltham, MA, United States), we determined the elemental composition (i.e., carbon, hydrogen, nitrogen, and sulfur content) of the biomass materials. The analysis was performed on dry samples fragmented into particle sizes of less than 0.2 mm, according to the PN-EN standard [28]. Table 4 lists the technical specifications of the device.

### 2.4. Measurement of the Exhaust Gas Composition

The exhaust gas composition was measured using a Wöhler A 550 analyzer. Individual compounds in the flue gas were detected using an electrochemical non-dispersive infrared sensor (NDIR). We first calibrated the analyzer, then measured the exhaust gas compounds after the combustion process had stabilized. The sampling location was the flue gas pipe just behind the boiler. Values were recorded every second over 6 h of continuous boiler operation. Table 5 summarizes the technical specification of the analyzer.

The experiment provided a comparison of the effect of selected chemical active substances on the combustion process of wheat bran and beet pulp pellets. The measured emission values were recalculated to a reference oxygen content of 10 vol.%.

### 2.5. Measurement of the Combustion Process Temperature

The temperature in the combustion chamber was measured according to the PN-EN standard. The temperature was recorded using three K-type thermocouples connected to the PAR AR205 recorder. The temperature was recorded every second for the entire period of the boiler operation. The mean measurement error in the results was ±1.5 °C.

## 3. Results

We conducted a series of tests to determine and compare the effects of selected catalytic additives on the combustion process of wheat bran and beet pulp pellets. The measured values of individual pollutant emissions were converted to a reference oxygen content (10 vol.% of content in the exhaust gas).

### 3.1. Biofuel Physicochemical Analysis

Table 6 and Table 7 present the results of our analysis of the agricultural origin raw materials.

Table 8 and Table 9 present the results of our elemental analysis of the wheat bran and beet pulp.

### 3.2. Analysis of the Exhaust Gas Composition

We compared our measurements with the figures for burning A1 class wood pellets (used as a reference, as in [29]), which are a popular type of energy carrier. Using the analyzer manufacturer’s software, the results were automatically converted into an oxygen content of 10 vol.% in the exhaust gases.

Table 10 provides a key to the terms used in the charts that indicate these results.

**Figure 4:**
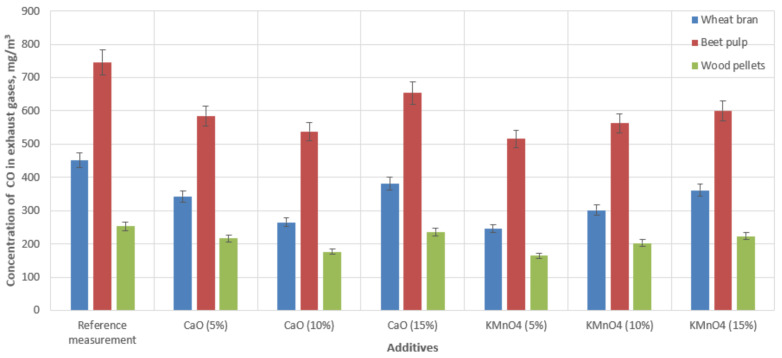
Concentrations of carbon monoxide in the flue gas of a low-power boiler while burning biomass with catalytic additives.

Figure 4 shows the relationship between the CO concentration in the exhaust gases and the type of biofuel and catalytic additive used in the experiment.

During the biomass waste combustion process, the CO emissions reached levels of 451 mg‧m^−3^ for wheat bran pellets and 746 mg‧m^−3^ for beet pulp pellets. Carbon monoxide emissions during the combustion of commercial fuel (wood pellets) reached a level of 252 mg‧m^−3^. The use of additives in combination with fuel during combustion reduced the CO concentration in the exhaust gases in each case. During the combustion of wheat bran pellets, carbon monoxide emissions decreased, on average, by 15–41% using a CaO-based additive and 20–45% using a KMnO_4_-based additive. When burning the beet pulp pellets, the CO emissions were reduced by 12–21% using a CaO-based additive, depending on the additive concentration, and 19–30% using a KMnO_4_-based additive, also depending on the concentration of the active substance. While feeding the boiler with wood pellets, the CO emissions into the atmosphere were reduced by 8–18% using CaO, depending on the concentration of the substance, and 3.5–30% using KMnO_4_. These data were the average values of two measurement sets.

**Figure 5:**
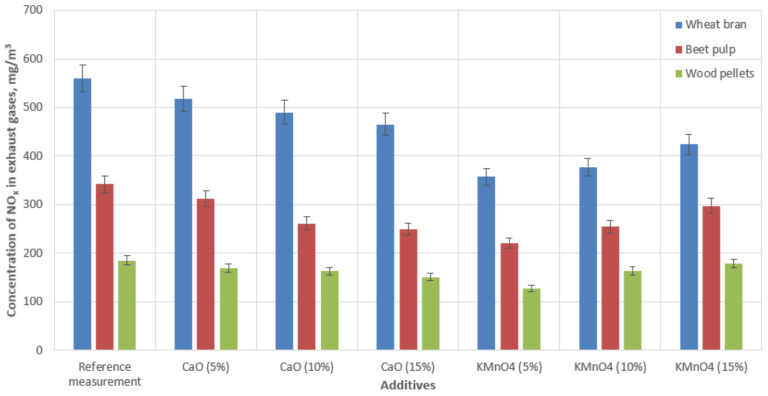
Concentrations of nitrogen oxides in the flue gas of a low-power boiler during the process of burning biomass with catalytic additives.

Figure 5 indicates the relationship between the exhaust gas NO_x_ concentration, the type of biofuel, and the catalyst.

We recorded the concentrations of nitrogen oxides emitted during the combustion of the biomass pellets. NO_x_ emissions varied around 559 mg‧m^−3^ during the combustion of wheat bran pellets, 341 mg‧m^−3^ in the case of beet pulp pellets, and 185 mg‧m^−3^ for wood pellets. The addition of the CaO-based additive reduced the NO_x_ emissions by 7–16% (wheat bran pellets), 8–27% (beet pulp pellets), and 8–18% (wood pellets), depending on the concentration of the substance. Burning the biomass with the KMnO_4_-based additives also succeeded in reducing the concentrations of nitrogen oxides in the flue gas. Using this additive, NO_x_ emissions were reduced by 24–36% (wheat bran), 12–35% (beet pulp), and 3.5–30% (wood pellets), depending on the concentration of the active substance. These data were the average values of two measurement sets.

**Figure 6:**
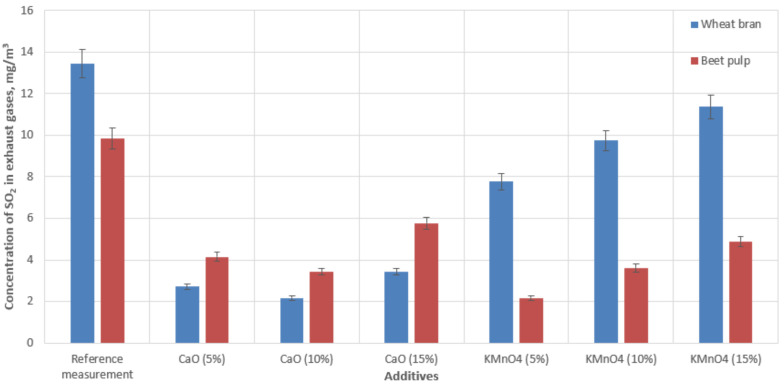
Concentrations of sulfur oxides in the flue gas of a low-power boiler during the process of burning biomass with catalytic additives.

Figure 6 indicates the effect of the biofuel and catalytic additives on the concentrations of sulfur dioxide (SO_2_) in the exhaust gases.

We also recorded reduced concentrations of SO_2_ during the process of burning wheat bran and beet pulp with additives. No reduction in the composition of exhaust gases was observed while burning wood pellets in the boiler. The SO_2_ emissions were 13.4 mg‧m^−3^ during the combustion of wheat bran pellets and 9.8 mg‧m^−3^ in the case of beet pulp pellets. Each of the additives in combination with the biomass fuel produced a reduction in SO_2_ concentration in the exhaust gases. While burning wheat bran, the SO_2_ emissions decreased by 74–83% using the CaO-based additive and 15–42% using the KMnO_4_-based additive, depending on the concentration of the active substance. In the case of burning beet pulp pellets, the SO_2_ emissions were reduced by 71–87% using the CaO-based additive and 41–65% using KMnO_4_, depending on the concentration of the additive. These data were the average values of two measurement sets.

### 3.3. Measurement of the Combustion Process Temperature

**Figure 7:**
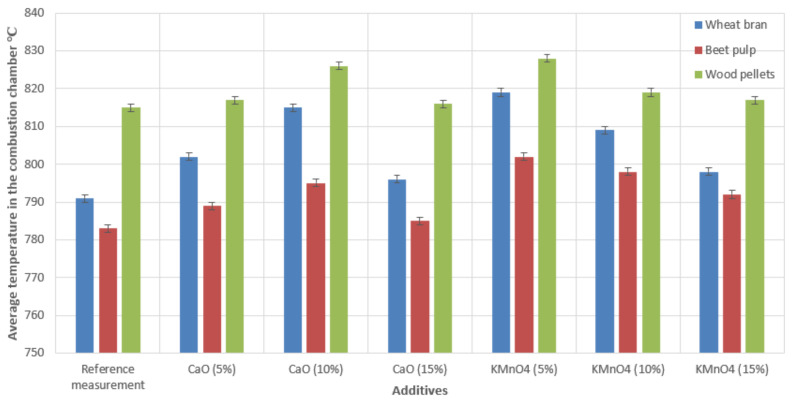
The distribution of the average temperatures in the combustion chamber during the combustion of biomass with catalytic additives.

Figure 7 presents the average temperature registered in the combustion chamber of the boiler after the stabilization of the combustion process.

We recorded increases in the average temperatures in the combustion chamber of the low-power boiler during the combustion of biomass fuels in combination with CaO and KMnO_4_ additives (in all concentrations). The average increase in the recorded temperatures in the boiler’s combustion chamber in the case of wheat bran pellets was 5 °C–24 °C depending on the CaO concentration and 7 °C–28 °C depending on KMnO_4_ concentration. In the case of beet pulp pellets, the temperature increased in the combustion chamber on average by 2 °C–12 °C with the addition of CaO, depending on its concentration, and 9 °C–19 °C with the addition of KMnO_4_, also depending on concentration. While feeding the boiler with the most popular biomass fuel, i.e., wood pellets, the temperature increased by 1 °C–11 °C using the CaO additive, varying with concentration, and 2 °C–13 °C while using the KMnO_4_ additive. These data were the average values of two measurement sets.

## 4. Discussion

This article’s subject matter is challenging to compare with the achievements published in other journals. The use of catalytic additives incinerated in combination with waste biomass in low-power boilers is a new approach to the problem of limiting emissions from combustion processes. The only similar studies were works that studied flue gas cleaning in large industrial power units. Due to the scale and different conditions in the combustion process in low-power boilers, these results cannot be used for making comparisons.

The catalysts (CaO and KMnO_4_) used in our experiment improved the combustion process and reduced the concentrations of pollutants found in the exhaust gases. Managed through the energy process, these substances compensated for the adverse properties of biomass waste. Due to the high quantities of nitrogen and sulfur in the main mixture, the amount of emissions of nitrogen compounds during combustion was significant. According to the fuel mechanism of the formation of nitrogen oxides [30], high nitrogen content in biomass results in increased NO_x_ emissions in exhaust gases. The use of catalytic additives in combination with fuel during the combustion process produced large reductions in emissions of these gases; in the best variants, CaO achieved a 41% reduction, and KMnO_4_ achieved a 45% reduction. Sulfur contained in the two biomass wastes (wheat bran and beet pulp) produced SO_2_ during the combustion process. The use of additives in combination with these materials during combustion reduced the sulfur oxide emissions into the atmosphere. In the best variants, the addition of CaO reduced SO_2_ emissions by 83%. The addition of KMnO_4_ produced the greatest reduction of SO_2_ pollution, achieving slightly over 87%.

The use of catalytic additives increased the average temperature in the combustion chamber by 1 °C–13 °C, depending on the concentration and type of additive. By using catalytic additives, the combustion process was more efficient as a result of improvement in the reaction toward complete combustion. Therefore, the temperatures increased and the process reduced the concentrations of CO emissions. In the best variant, the addition of CaO reduced the CO emissions by 41%, and similarly, the addition of KMnO_4_ to the biomass pellets reduced the CO emissions by 45%. Improvement in the quality of combustion and consequent burning of flammable components in the exhaust gases increased the overall efficiency of the combustion process and reduced the chimney loss. 

A comparison of the results in Figure 3, Figure 4, Figure 5 and Figure 6 indicated that the reduction in emissions was not always directly dependent on the quantity of the additive used. The addition of 10% CaO led to the largest reductions in CO and SO_2_ emissions; however, a 15% addition to the combusted fuels produced the best reduction in NO_x_ emissions. In the case of KMnO_4_, a 5% addition in all cases achieved the largest emissions reduction. A comparison of the results in Figure 6 indicated that temperature correlated with the quantity of decreased emissions. We also observed that the emissions reduction and the efficiency of the entire combustion process were more significant at higher temperatures, (see Figure 3, referring to CO emissions). The results also revealed that some additive quantities were too large, and consequently, the combustion process was not as efficient. However, it should be noted that all additives had a positive effect on reducing emissions (compared with emissions produced by burning raw materials only).

During the combustion of biomass waste in combination with the KMnO_4_ additive, slight sintering of burned material occurred and may have slightly affected the combustion process. When CaO was used as an additive, ash agglomeration did not occur. This positive effect from CaO was also verified in the work [31]; the ability of CaO to prevent sintering is a great advantage in obtaining a proper combustion process.

The use of catalytic additives positively affects the combustion process and reduces the emission of harmful substances into the atmosphere. The catalytic additives applied in the current study compensated for the adverse properties encountered in the waste materials from the food production and processing industry. Catalytic additives enable the use of waste as energy carriers in the incineration process. The widespread use of CaO and KMnO_4_ additives in other biomass waste treatments beyond only storage or neutralization would potentially benefit a large number of food-processing companies in managing their waste energy. In this way, manufacturers may be able to cover their heat requirements in whole or partially at no significant cost. The use of catalytic additives has a positive effect on the environment and the economy, and to some extent, enables the replacement of fossil fuels with ecological organic fuels.

## 5. Conclusions

The findings in other articles indicate that catalytic additives are a suitable technology for treating exhaust gases, including gases produced during the combustion of biomass waste. The main scientific challenge is determining the correct balance of catalytic compounds to combine high reduction efficiency with the low cost of the selected substance. The aim of the current study was to identify a suitable catalyst for the combustion of wheat bran and beet pulp biomass waste. The approach we proposed is innovative. To the best of our knowledge, no other research team has yet investigated the effect of CaO and KMnO_4_ catalytic additives on the quality of exhaust gases during the process of burning wheat bran and beet pulp.

From our research, we concluded that the use of catalytic additives in combination with biomass waste during the combustion process eliminated some of the adverse physicochemical properties of these materials. The use of this type of waste in energy production processes, including heat produced via direct combustion, is therefore possible and economically reasonable. The use of catalytic additives reduces the emission of harmful substances, increases the boiler’s efficiency, and reduces the consumption of biomass fuel.

Similar analyses were done on other catalytic additives used during the combustion of wood pellets [32] and sunflower husks [33]. Another study investigated the use of urea to reduce NO_x_ emissions [34] from low-power biomass boilers. These catalytic additives reduced emissions effectively, and the profitability of their use increased as a consequence of reduced fuel consumption and increased combustion efficiency. Urea is also effective [22], but the degree of emissions reduction relates to the optimal temperature in the combustion chamber.

The research describes a prospectively useful solution for entrepreneurs who produce this type of biomass waste in the technological processes of their food processing endeavors. However, the use of catalytic additives has some limitations. The active substance of the catalytic system is characterized by the most effective action in a specific temperature window. Too low or too high of a temperature may diminish its efficiency. Future research in this area could investigate the effect of catalytic additives on boiler efficiency, determine the effect of burning substances in combination on the lifespan of the heating surfaces in the boiler, the effect of additives on the composition of the ash resulting from the combustion process, or identify new types of catalytic additives (motivated by the need to increase the efficiency of flue gas cleaning). We plan to study, compare, and verify the results of using smaller additive quantities, for example, 0.5–5.0%, and investigate the effect of catalytic systems and other organic biomass waste materials on flue gas quality.

## Figures and Tables

**Table 1:** Specifications of the VARIANT SL33A pellet boiler.

Parameter	Unit	Value
Boiler class according to Ecodesign Directive	-	4
Boiler output	kW	31.5
Boiler efficiency	%	87.3
Boiler weight	kg	320
Maximum operating temperature	°C	90
Minimum return water temperature	°C	70
Fuel tank capacity	dm^3^	210
Highest working overpressure	bar	2

**Table 2:** Technical specification of TGA 701 analyzer.

Parameter	Unit	Value
Sample mass	g	1
Number of samples	pcs	19 (+1 reference)
Precision	%	0.02
Temperature control range	°C	100–1000
Temperature control accuracy	°C	±2
Temperature control stability	°C	±2
Maximum ramp rate from ambient to 104 °C	°C·min^−1^	15
Maximum ramp rate from 104 °C to 1000 °C	°C·min^−1^	50
Gas pressure	bar	3.1 for air, 2.4 for nitrogen, 2.4 for oxygen
Minimum gas purity	%	99.9 for nitrogen, 99.5 for oxygen

**Table 3:** Technical specifications of the IKA C 200 calorimeter.

Parameter	Unit	Value
Maximum output energy	J	40,000
Temperature sensor resolution	°C	0.0001
Oxygen working pressure	bar	40
Initial temperature settings	°C	18–25

**Table 4:** Technical specifications of the PerkinElmer CHNS/0 2400 analyzer.

Parameter	Unit	Value
Temperature range	°C	100–1100
Sample size	mg	0–500
Accuracy	%	≤0.3
Carbon analytical range	mg	0.001–3.6
Hydrogen analytical range	mg	0.001–1.0
Nitrogen analytical range	mg	0.001–6.0
Sulphur analytical range	mg	0.001–2.0
Oxygen analytical range	mg	0.001–2.0

**Table 5:** Technical specifications of the Wöhler A 550 flue gas analyzer.

Component	Measurement Principle	Range	Accuracy
O_2_	Electrochemical sensor	0–21 vol.%	±0.3% vol.%
CO	Electrochemical sensor, H_2_ compensated	0–4000 vol. ppm	±20 ppm (< 400 ppm), otherwise ±5% of measurement
CO_2_	NDIR	0–40%	±0.3 vol.% (0–6 vol.%) otherwise ±5% of reading
NO	Electrochemical sensor	0–3000 vol. ppm (continuously up to 1000)	±5 vol. ppm (<100 ppm), otherwise 5% of reading
NO_x_	Electrochemical sensor	0–1000 vol. ppm (continuously up to 200)	±5 vol. ppm (<100 ppm), otherwise 5% of reading
SO_2_	Electrochemical sensor	0–5000 vol. ppm	±10 vol. ppm (0–200 ppm), otherwise 5% of reading

**Table 6:** Results of the analysis of wheat bran.

Parameter	Unit	Value
Moisture content	%	12.73 ± 1.91
Ash content	%	5.65 ± 0.85
Volatile matter content	%	65.27 ± 11.75
Higher heating value	MJ·kg^−1^	16.92 ± 1.69
Lower heating value	MJ·kg^−1^	13.19 ± 1.32

**Table 7:** Results of the analysis of beet pulp.

Parameter	Unit	Value
Moisture content	%	11.81 ± 1.77
Ash content	%	7.14 ± 1.07
Volatile matter content	%	66.15 ± 11.91
Higher heating value	MJ·kg^−1^	15.04 ± 1.50
Lower heating value	MJ·kg^−1^	11.79 ± 1.18

**Table 8:** Results of the elemental analysis of wheat bran.

Parameter	Unit	Value
Nitrogen content	%	2.70 ± 0.40
Carbon content	%	40.50 ± 6.08
Hydrogen content	%	6.67 ± 1.00
Sulfur content	%	0.16 ± 0.02

**Table 9:** Results of the elemental analysis of beet pulp.

Parameter	Unit	Value
Nitrogen content	%	1.34 ± 0.20
Carbon content	%	37.90 ± 5.68
Hydrogen content	%	6.18 ± 0.93
Sulfur content	%	0.16 ± 0.02

**Table 10:** Terms used in the charts.

Abbreviation	Description
RM	Reference measurement
CaO (5%)	5% CaO content in relation to the mass of fuel burned
CaO (10%)	10% CaO content in relation to the mass of fuel burned
CaO (15%)	15% CaO content in relation to the mass of fuel burned
KMnO_4_ (5%)	5% KMnO_4_ content in relation to the mass of fuel burned
KMnO_4_ (10%)	10% KMnO_4_ content in relation to the mass of fuel burned
KMnO_4_ (15%)	15% KMnO_4_ content in relation to the mass of fuel burned

## Data Availability

The data presented in this study are available on request from the corresponding author.
